# Assessment of nutritional status and health behaviors in yoga-trained women versus exercisers

**DOI:** 10.3389/fnut.2024.1334428

**Published:** 2024-04-30

**Authors:** Anna Gogojewicz, Łucja Pilaczyńska-Szcześniak, Natalia Popierz-Rydlewska, Patxi León-Guereño, Ewa Malchrowicz-Mośko

**Affiliations:** ^1^Department of Food and Nutrition, Faculty of Health Sciences, Poznan University of Physical Education, Poznań, Poland; ^2^Department of Physiotherapy, Faculty of Medicine and Health Sciences, University of Kalisz, Kalisz, Poland; ^3^Health, Physical Activity and Sports Science Laboratory, Department of Physical Activity and Sports, Faculty of Education and Sport, University of Deusto, Bilbao, Spain; ^4^Department of Sports Tourism, Faculty of Sport Sciences, Poznan University of Physical Education, Poznan, Poland

**Keywords:** yoga, health behaviors, nutritional status, physical activity, healthy lifestyle

## Abstract

**Introduction:**

Recreational physical activity is becoming more popular due to the increased public awareness about the beneficial effects on health status and quality of life. The aim of the study was to assess the nutritional status and health behaviors of women who regularly practice yoga as a form of physical recreation and to compare them with those who had not practiced before and had just signed up for yoga classes. A total of 143 women took part in this study.

**Methods:**

The nutritional status was assessed based on the obtained anthropometric measurements. The following indicators were calculated: Body Mass Index (BMI) and waist-to-hip ratio (WHR), determining the visceral accumulation of fat tissue. Health behaviors were assessed using a standardized five-point scale Health Behavior Inventory (HBI).

**Results:**

There were significant differences in the value of the general health behavior index, the sten scale, and the subscale regarding proper eating habits in the study groups compared to women who had not practiced yoga before (control group). Health behaviors indicators, particularly proper eating habits, are significantly higher in women participating regularly in yoga exercises, indicating a higher awareness among yoga practitioners.

**Conclusion:**

It can be suggested that yoga participation as a recreational physical activity can be an appropriate option for pursuing healthy habits.

## 1 Introduction

Recreational forms of physical activity are becoming very popular (1–3). There is increasing awareness of the beneficial impact of physical activity on mental and physical health and on improving the quality of life. In recent years, the number of people practicing it regularly has increased due to its health benefits (4–7). Lifestyle and related health behaviors are important factors determining health (8). Health behavior is any intentional action taken by an individual whose aim is to maintain or increase health potential, regardless of its effectiveness (9). Health behaviors cause specific positive or negative health effects in people implementing them (10). Personal habits related to physical activity, healthy habits, healthy eating and motivation are of interest to contemporary researchers (11). In recent years, yoga has been one of the most popular forms of physical activity. Yoga is an ancient Indian system of philosophy, a mind–body discipline encompassing an array of philosophical precepts, mental attitudes, and physical practice. The most popular hatha yoga covers many different styles. In the most popular lyengar or Ashtanga practitioners in asanas focus on the detailed positioning of bodies. Both styles of yoga are rich in asanas, and ashtanga also focuses on breathing control during practice (12). Research from other authors confirms the pleiotropic effect of yoga on the human body, including the circulatory system, and alleviates the effects of diseases of the musculoskeletal system (13–18). Yoga improves lung function, strength of respiratory and expiratory muscles as well as skeletal muscle strength and endurance (19). Yoga is also recommended for patients with non-communicable disease such as cancer (20). Currently, it is also one of the most frequently used therapies for people with depressive disorders (21).

The role of yoga in ecology and nutrition for sustainable healthy living is very important as well (22, 23). Yoga can support overweight people in eating healthier and increasing physical activity, which ultimately leads to a lower BMI (24). Chin-Cheng et al. (25) indicated in the studies among students in Yogalates class the effectiveness of multimedia-assisted learning. Perhaps it is worth knowing that this type of teaching model is included in the physical education programs, which influenced the use of functions after inclusion and the formation of activity habits (25). Yoga can also be an excellent form of activity in improving the physical fitness of children in early childhood (26). Studies suggest that yoga participation can be associated with mindful eating and the adoption and maintenance of other positive health-related outcomes such as regular physical activity and weight management (27). The results of the Watts et al. (28) study confirm that yoga is a promising intervention aimed at improving the health of overweight people. Regular yoga practice can support the development of physical activity habits and healthy eating patterns, which in turn is associated with greater body awareness (28).

At the same time, however, yoga has also been criticized for sometimes overly expecting yoga practitioners to pay excessive attention to diet and food quality. Some studies suggest that yoga teachers should avoid excessive reference to a healthy diet, which is component of yoga practice (29). In Domingues and Carmo’s (30) study, interest in a healthy diet among the population of yoga practitioners was one of the strongest predictors of orthorexia nervosa. People who often control their body weight and use techniques common among yogis, such as cleansing, detox, fasting or vegetarian diets, are particularly susceptible to eating disorders (30). In a study conducted by Erkin and Gol (31) among yogis practicing in Turkey, it was found that the vast majority of yoga practitioners are at risk of orthorexia, and some factors, such as marital status and the presence of chronic diseases, significantly influence the tendency to mental orthorexia in yoga practitioners. Even though yoga provides several benefits, it is not clear whether the practice of yoga is associated with proper nutritional and health habits. Yoga can provide many health benefits, and studies comparing the effects of yoga and other forms of activity indicate that yoga may be as or even more effective than other physical activities in improving various indicators of health outcomes. Future research is therefore needed to examine the differences between yoga and other forms of physical activity in terms of their impact on physical and mental health (32).

Therefore, the objective of the present study is to assess the nutritional status and health behaviors of women practicing yoga as a form of physical recreation, and to compare them with those who had not practiced before and just signed up for yoga classes.

## 2 Materials and methods

### 2.1 Participants, design and procedure

The study involved women aged 30–59, members of yoga clubs and fitness clubs from the Greater Poland Voivodeship. Of the 143 respondents, 68 regularly practiced yoga and 75 had no previous experience with yoga. Women from the control group used other forms of recreation, such as walking, swimming, aerobics, aqua fitness and Nordic walking. Both groups had similar training experience and duration (at least 2 years). Both groups were homogeneous in terms of anthropometric characteristics. The respondents who participated in the study had been attending yoga classes or other kinds of classes (aerobic, Nordic Walking, etc.) at least twice a week regularly for 90 min for two years or more.

The yoga classes were conducted by qualified instructors. The leading style was Ashtanga Yoga, one of the forms of Hatha and Iyengar Yoga, the most popular in Poland. This practice is characterized by great attention to the precision of body positioning in the asana. Great importance is attached to proper breathing in positions. The Iyengar method uses many different yoga aids, e.g., blocks and straps, to help practitioners correctly position their bodies (33).

Yoga instructors provided their groups with information about the planned research. Leaflets describing the research were left at the places of classes. Interested persons received detailed information about the purpose and method of research carried out by the research supervisor. Participants were allowed to familiarize themselves with all testing procedures and provided written informed consent before the study. The study protocol was reviewed and approved by the Bioethics Committee at the Poznan University of Medical Sciences (reference number 824/10) and was performed in accordance with the Declaration of Helsinki.

### 2.2 Variables and instruments

#### 2.2.1 Nutritional status

Body weight and height were measured on an in a fasting state using a certified Radwag device (Radom, Poland) with an accuracy of 0.01 kg and in the case of body height 0.5 cm. Waist and hip circumference were measured according to World Health Organization (WHO) recommendations (34) using a non-stretchable measuring tape. Based on the obtained measurement results, the following indices were calculated: BMI and WHR. The BMI classification recommended by the WHO was used to interpret the results (34).

#### 2.2.2 Health behaviors

Health behaviors were assessed using the five-point scale Health Behavior Inventory (HBI) by Juczyński (35). This questionnaire is a Polish, standardized tool for measuring the overall intensity of health practices. Questionnaire contains 24 statements describing health-related behaviors, which are divided into 4 categories: proper eating habits, preventive behaviors, health practices and positive mental attitude. The statements were assessed, respectively, using a five-point response scale: 1-almost never, 2-rarely, 3-from time to time, 4-often, 5-almost always. The obtained points were summed up. The general index of the intensity of health behaviors measured by the HBI scale ranges from 24 to 120 points. The higher the score, the greater the intensity of the declared health behaviors. The obtained number of points was converted to the sten scale. Low scores are (1–4 sten), average (5–6 sten) and high (7–10 sten). The reliability of the HBI is Cronbach’s α = 0.85, and its four subscales range from 0.60 to 0.65.

### 2.3 Statistical analysis

The obtained data were subjected to statistical analysis using the Statistica version 13.3 (TIBCO Software Inc., Palo Alto, CA, United States). Data are presented as means, standard deviations (SD), minimum and maximum (min ÷ max). The Shapiro–Wilk test was used to check the data for normal distribution. Comparisons of normally distributed variables between the two groups were assessed using Student’s t test and the Mann–Whitney test was used for non-normally distributed variables. A *p*-value <0.05 was considered significant.

## 3 Results

Table 1 shows a comparative analysis of anthropometric characteristics indicates the lack of statistically significant differences, which proves the high similarity of both groups in terms of somatic structure. All women declared good health condition.

**Table 1:** Descriptive statistics of somatic parameters.

Variables	Yoga group (*n* = 68)	Control group (*n* = 75)	*p*-value
	x ± SD (min ÷ max)		
Height (cm)	164.6 ± 6.40 (154 ÷ 180)	165.6 ± 5.59 (153 ÷ 179)	0.2139
Body mass (kg)	61.9 ± 7.01 (50 ÷ 80)	63.3 ± 7.89 (50 ÷ 81)	0.4188
BMI (kg/m^2^)	22.9 ± 2.43 (18.6 ÷ 28.5)	23.1 ± 2.83 (18.8 ÷ 29.7)	0.7648
Waist circumference (cm)	78.0 ± 6.85 (65 ÷ 93)	76.6 ± 8.19 (59 ÷ 102)	0.0501
Hip circumference (cm)	100.0 ± 6.21 (90 ÷ 116)	98.6 ± 6.66 (82 ÷ 112)	0.1719
WHR	0.79 ± 0.05 (0.66 ÷ 0.92)	0.78 ± 0.06 (0.67 ÷ 0.93)	0.1046

Data are presented as mean ± SD, minimum and maximum. BMI – body mass index; WHR – waist-to-hip-ratio.

The average value of BMI and WHR for the study and control groups is within the reference values. However, the analysis of the distribution of BMI values revealed that in the assessed groups there were women whose BMI exceeded 25 kg/m^2^.

The research results showed that study groups’ the average health behaviors and proper eating habits differ significantly between the study groups (Table 2). Women who regularly participate in yoga classes are characterized by a significantly higher value of the health behavior index, the raw score of which for the study group was on average 90.2 ± 12.74 points, and in the control group 84.7 ± 10.82 (*p* < 0.01). Proper eating habits are significantly higher in the group of women participating regularly in yoga exercises as well.

**Table 2:** Set of behavior health traits (BHT) of the studied and control group.

Variables	Yoga group (*n* = 68)	Control group (*n* = 75)	*p*-value
	x ± SD (min ÷ max)		
Health behavior index	90.2 ± 12.74 (62 ÷ 119)	84.7 ± 10.82 (61 ÷ 103)	**0.0169**
Positive mental attitude	3.82 ± 0.681 (2 ÷ 5)	3.72 ± 0.665 (1.84 ÷ 5)	0.4306
Preventive behaviors	3.76 ± 0.639 (2,17 ÷ 5)	3.59 ± 0.63 (1.67 ÷ 4.84)	0.1363
Proper eating habits	3.98 ± 0.620 (2,34 ÷ 5)	3.53 ± 0.696 (1.67 ÷ 5)	**0.0001**
Health practices	3.49 ± 0.675 (1.67 ÷ 5)	3.29 ± 0,62 (1.84 ÷ 4.67)	0.1332

Data are presented as mean ± SD, minimum and maximum. Statistically significant.

The results presented in Table 2 show that although the group of women attending yoga classes regularly obtained higher results in all tested subscales compared to the control group, significant differences were observed only in the case of proper eating habits.

**Figure 1:**
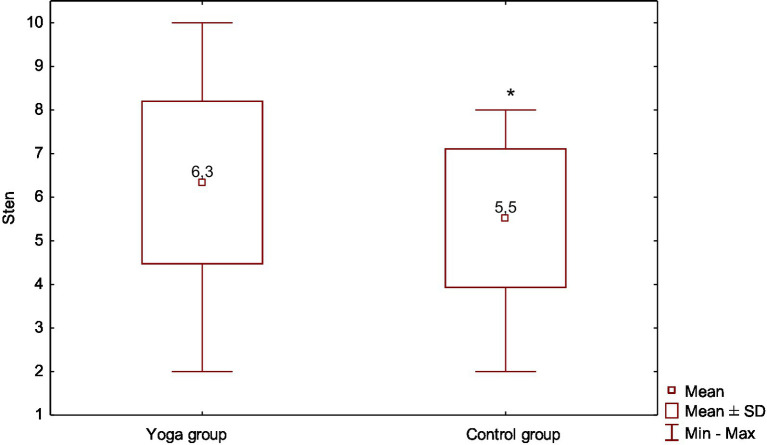
Comparative analysis of the average values of standard sten test in the studied groups. Data are presented as mean ± SD, minimum and maximum.

The research results indicate that in the group of women exercising yoga regularly, health behaviors assessed with the sten scale turned out to be significantly higher (*p* = 0.0177) compared to the control group (Figure 1).

## 4 Discussion

The aim of the study was to assess the nutritional status and health behaviors of women who regularly practice yoga as a form of physical recreation, and to compare them with those who had not practiced it before. The average value of BMI and WHR for the study and control groups resulted within the reference values. However, the analysis of the distribution of BMI values revealed that in the assessed groups there were women whose BMI exceeded 25 kg/m^2^. According to Gokal et al. (36), yoga exercises may be recommended to normalize BMI and WHR in overweight and obese people. They can also be used in the treatment of diabetes, hypertension and other metabolic diseases. This is also confirmed by the research of Swapna et al. (37), in which obese patients with type 2 diabetes participating in an intensive, weekly yoga course achieved a decrease in body weight by 2.15%, BMI by 2.1%, and WHR by 4.4%.

The results of our research are consistent with the study of Lim and Hyun (38). In their opinion, yoga helps shape healthy behaviors in yogis and induces positive beliefs about their subjective health, thus triggering a cycle of positive reinforcement. There are reports suggesting the implementation of low- and moderate-intensity yoga classes also among the elderly population to prevent physical and mental health (39). Also, research conducted in Bulgaria with the participation of 89 women practicing yoga showed that yoga practitioners eat healthily – the respondents consumed small amounts of pork and beef, and 60% did not eat meat at all. They consumed an average of 600 g of fruit and vegetables per day, corresponding to the 400 g recommended by the WHO. Practicing yoga helps maintain a proper body mass, which is one of the conditions for a healthy lifestyle. Assessment of the nutritional status of yoga participants was consistent with the WHO and American Cancer Society recommendations for a healthy diet (40).

According to Sreedevi et al. (41), a conscious sustained effort practiced through attitudinal changes implemented on good food habits and choices, exercise, yoga and meditation may have a cumulative impact on the continued beneficial effect on health and overall well-being.

Our results showed that women practicing yoga had a higher rate of proper eating habits. This is probably the result of obtaining higher scores on one of the four subscales regarding proper nutrition. Moreover, there were no significant differences in the nutritional status of women practicing yoga compared to women who did not. We suppose this is most likely due to the benefits of physical activity. Therefore, yoga seems to be a complex intervention that also covers issues related to achieving an ethical and healthy lifestyle, such as consciously making healthy and ethical food choices (42).

Yoga comes from religious and spiritual traditions, so in this case, particular emphasis is placed on a hygienic lifestyle consistent with health practices. In addition, some varieties of Yoga assume a vegetarian diet associated with nonviolence towards living beings. Yoga has connections with Hinduism, Buddhism, and Jainism based on a shared philosophical framework of oneness with all beings and a belief in ahimsa, or nonviolence. Therefore, changes in health behaviors among yoga practitioners seem to be more frequent and longer lasting than among other groups of physically active people practicing exercises unrelated to religious beliefs (43).

However, even recent investigations try to show the effects of Yoga as a recreational physical activity (44), and despite the findings obtained in this investigation, further research is needed.

## 5 Limitations of the study and future study suggestions

Even though the objectives of this research have been answered, the study has some limitations. On the one hand, based on the research conducted, it is difficult to claim whether this was due to yoga exercises or whether this group consisted of women who cared more about their health in general. On the other hand, a number of participants and a more significant sample would be needed to generalize the obtained results. Moreover, data were collected based on self-report, which may cause bias. In turn, if detailed information about participants’ yoga and exercise habits is not collected, this may also pose a limitation in the interpretation of the results. Other than that, for future research, male yoga practitioners should be taken into account to show wider and more generalizable results and to see whether the benefits are the same gender-wise. The questionnaire used in our study is a Polish standardized tool for measuring the overall intensity of health practices. The reliability of the HBI, Cronbach’s alpha is 0.85, and its four subscales range from 0.60 to 0.65, for which the values calculated for Cronbach’s alpha are near or below the acceptable values. We suppose this is mainly due to the small number of items contributing to the subscales. Perhaps slightly increasing the number of items would lead to acceptable values for Cronbach’s alpha.

## 6 Conclusion

To summarize the obtained research results, it could be suggested that the index of health behaviors and proper eating habits is significantly higher in the group of women who regularly participate in yoga classes. From these results, indicators of healthy habits in daily life could be associated with the regular practice of yoga activities. Therefore, yoga might be a good recreational physical activity to try to pursue health from a public health perspective.

## Data availability statement

The raw data supporting the conclusions of this article will be made available by the authors, without undue reservation.

## Ethics statement

The study was reviewed and approved by Bioethics Committee at Poznan University of Medical Sciences in Poland (number 824/10). Written informed consent to participate in this study was provided by the participants.

## Author contributions

AG: Data curation, Formal analysis, Investigation, Resources, Writing – original draft, Writing – review & editing. ŁP-S: Methodology, Project administration, Supervision, Writing – review & editing. NP-R: Formal analysis, Visualization, Writing – review & editing. PL-G: Software, Writing – review & editing. EM-M: Resources, Writing – original draft.
