# Supplementary material for: Assessment of nutritional status and health behaviors in yoga-trained women versus exercisers
Source: Front Nutr. 2024 Apr 30;11:1334428. doi: 10.3389/fnut.2024.1334428 (PMC11091242; doi:10.3389/fnut.2024.1334428)
Supplement: Supplementary file 1 [file Data_Sheet_1.pdf]

Appendix 1: Z. Juczyński:

Gender/Age:

How often during the year do you engage in the behaviors listed below?

1- almost never
2- rarely
3- from time to time
4- ofter
5- almost always

1. I eat a lot of fruits and vegetables
2. I avoid colds
3. I take seriously the advice of people who express concern about my health
4. I get enough rest
5. I limit the consumption of products such as animal fats and sugar
6. I have written down the telephone numbers of the emergency services
7. I avoid situations that have a depressing effect on me
8. I avoid overwork
9. I care about proper nutrition
10. I follow medical recommendations resulting from my tests
11. I try to avoid strong emotions, stress and tension
12. I control my body weight
13. I avoid eating food with preservatives
14. I regularly undergo medical examinations
15. I have friends and a settled family life
16. I sleep enough
17. I avoid salt and heavily salted foods
18. I try to learn how others avoid illness
19. I avoid feelings such as anger, anxiety and depression
20. I limit smoking
21. I eat whole grain bread
22. I try to obtain medical information and understand the causes of health and disease
23. I think positively

24. I avoid excessive physical exercise

25. Other…

ZZ	sten	PN	ZP	PN	PZ
